# Factors Influencing Undesirable Sensory Properties in Protein Hydrolysates and Remediation Strategies for Food Applications

**DOI:** 10.1111/1750-3841.71031

**Published:** 2026-03-29

**Authors:** Tejaswini R. B. Ramakrishna, Tone Aspevik, Birthe Vang, Kjersti Lian, Charlotte Jacobsen, Volha Shapaval, Pål Puntervoll, Gro E. K. Bjerga

**Affiliations:** ^1^ Department of Biotechnology and Circular Economy NORCE Research Bergen Norway; ^2^ Department of Nutrition & Feed Technology Nofima Bergen Norway; ^3^ Department of Marine Biotechnology Nofima Tromsø Norway; ^4^ Research Group for Bioactives—Analysis and Application, National Food Institute Technical University of Denmark Lyngby Denmark; ^5^ Faculty of Science and Technology Norwegian University of Life Sciences Ås Norway

**Keywords:** enzymatic hydrolysis, odor, protein hydrolysates, protein hydrolysis, sensory properties, smell, taste

## Abstract

Enzymatic hydrolysis is used to produce protein hydrolysates from animal‐ and plant‐based sources, which are widely utilized in industry due to their nutritional, functional, and bioactive properties. However, these hydrolysates can exhibit unattractive sensory properties, such as off‐flavors or off‐odors, which limit their use in food products. The sensory properties of protein hydrolysates may stem from inherent attributes in the raw material or develop during processing—either as an intended consequence of hydrolysis or as an unintended side effect. This review summarizes key factors contributing to undesirable sensory attributes, and highlights processing steps influencing sensory properties, drawing on examples from plant‐ and animal‐sourced protein hydrolysates. Subsequently, an assessment of recent strategies to improve sensory properties is provided. While earlier research has mainly addressed bitterness, other undesirable sensory issues remain underexplored. Several factors affect sensory properties in protein hydrolysates during pre‐, peri‐, and post‐hydrolysis, such as choice of raw material (composition and freshness), process conditions, and microbial action. Through literature searches, 10 strategies for optimizing the sensory properties in protein hydrolysates have been identified: pretreatment of raw materials, application of antioxidants, enzymatic modification methods, use of Maillard reaction, fermentation processing techniques, solvent‐assisted extraction, adsorption removal techniques, membrane filtration processes, microencapsulation technology, and use of masking agents. While these strategies may make the protein hydrolysates more palatable and acceptable for consumption, their shortcomings include additional costs, time‐consuming processes, reduced nutritional value, and potential non‐compliance with food‐grade standards.

## 1 Introduction

Globally, the food industries generate about 1.6 billion tonnes of side streams annually (FAO 2013), often disposed of at high cost or deposited in landfills or elsewhere in the environment. This waste contributes to an estimated 3.3 billion tonnes of CO_2_ equivalent of greenhouse gases each year (FAO 2013). The growing risk of food scarcity is a significant concern, especially with the global population projected to reach 9.5 billion by 2050 (Henchion et al. 2017). Hence, an effective food management system is necessary to reduce the environmental impact and meet the food demand from an increasing population. The demand for protein‐rich diets is growing due to urbanization, rising incomes, and increasing consumer awareness of eating healthy foods (Henchion et al. 2017). In 2024, the market value of plant‐based protein was valued at approximately USD 10 billion, ocean‐based protein (from fisheries and aquaculture) was around USD 215 billion, and meat‐based protein exceeded USD 1 trillion, reflecting substantial variation in consumer preferences across diverse dietary sources (Shahbandeh 2025; Verified Mark. Rep. 2025; Market.Us 2025). During food processing of crops, livestock, and aquatic species, side streams are generated, which are not the main product, for example, unused parts of the crop plant (e.g., bran, straw, stem, and leaves), animal off‐cuts (e.g., head, bones, and viscera), skin, feathers, and blood (Xiao Sun et al. 2024; Jayathilakan et al. 2012). These materials are often underutilized or discarded, even though some of these side streams contain valuable nutrients such as protein, fats, and fibers, making them ideal for valorization.

Protein‐rich side streams contain substantial amounts of proteins, along with varying levels of lipids, carbohydrates or dietary fibers, minerals, and other bioactive compounds (Kristinsson and Rasco 2000; Czelej et al. 2022), although the proportions differ between plant‐, animal‐, and marine‐derived materials. Despite this nutrient content, these streams are often processed into compost, animal feed, or low‐value food products, and are typically sold at prices far below those of the primary products. A common strategy for increasing the market value of these side streams is to valorize them through protein hydrolysis. This involves applying chemical or enzymatic hydrolysis to break down the proteins into a mixture of peptides and amino acids, referred to as protein hydrolysates (Petrova et al. 2018). This review focuses on enzymatic hydrolysis, which is more specific, eco‐friendly, and gentler than traditional chemical treatments (Petrova et al. 2018). Importantly, the enzymatic treatment preserves essential amino acids without inducing racemization, thereby ensuring food safety and maintaining the nutritional value in the products (Petrova et al. 2018; Clemente 2000).

Enzymatic protein hydrolysis usually consists of five main steps: homogenizing the raw material; controlled enzymatic hydrolysis (Clemente 2000); termination of the enzymatic reaction (Le Maux et al. 2018; Nemati et al. 2024); fractionation of the mixture into water‐soluble, insoluble, and oil phases (Kristinsson and Rasco 2000); and final refinement of the peptide‐containing water‐soluble fraction through filtration and drying (Pasupuleti and Braun 2010). The resulting protein hydrolysates are used in high‐value sectors such as pharmaceuticals, food, feed, and other high‐value industrial applications (Davis et al. 2017; Honrado et al. 2024). However, protein hydrolysates often pose unique challenges such as bitterness, off‐flavors, and off‐odors, which may compromise consumer acceptance and their economic value. This review provides an overview of the main factors contributing to the development of sensory properties of protein hydrolysates derived from plant‐, animal‐, and marine‐sources, and it presents existing and emerging strategies to remediate undesirable taste and odor attributes in protein hydrolysates.

## 2 Methods

The scientific literature that forms the basis for this review was compiled using search engines like PubMed, Google Scholar, Elicit.ai, and targeted journals, covering the period from 2000 to 2025 in the English language. The initial search focused on strategies for remediating the sensory properties of protein hydrolysates, using keywords such as “protein hydrolysates taste and/or odor,” which yielded over 20,000 records. The search strategy was refined by incorporating additional terms, including “protein hydrolysates AND pre‐treatment/lipid‐oxidation/Maillard‐reaction/antioxidant/fermentation/micro‐encapsulation/membrane‐filtration/enzyme‐treatment/masking/adsorption,” along with terms related to plant and animal proteins. Search was further supplemented by consulting additional literature, including publications prior to 2000, to ensure comprehensive coverage of the topic. During the screening phase, titles and abstracts were initially assessed to identify studies of interest, followed by full‐text evaluation to exclude irrelevant papers.

## 3 Known Contributors to the Sensory Attributes of Protein Hydrolysates

Protein hydrolysates exhibit a wide range of sensory attributes, including desirable tastes such as umami, savory, or meaty notes (Schlichtherle‐Cerny and Amadò 2002; Fuke and Shimizu 1993; Su et al. 2012), and undesirable notes like bitterness, rancid, and metallic flavors, as well as odors ranging from pleasant to fishy, sulfurous, or ammonia‐like (Calkins and Hodgen 2007; Domínguez et al. 2019; Damodaran and Arora 2013; Caimeng Zhang et al. 2020; Goris et al. 2020; Yu Fu et al. 2019). These sensory perceptions arise from a complex mixture of non‐volatile and volatile compounds present in the protein hydrolysate. Some of these compounds originate from non‐protein components that remain in the water‐soluble fraction after fractionation and filtration (Domínguez et al. 2019). Non‐volatile compounds such as peptides, amino acids, and biogenic amines primarily contribute to taste attributes, whereas volatile compounds, including aldehydes, ketones, alcohols, sulfur‐containing molecules, and other low‐molecular‐weight metabolites (including semi‐volatile biogenic amines), largely dictate odor (Yu Fu et al. 2019; Caimeng Zhang et al. 2020).

### 3.1 Non‐Volatile Compounds: Taste

Free glutamic acid, and to a lesser extent free aspartic acid, together with peptides enriched in glutamic acid, contribute to an umami flavor in protein hydrolysates, a taste quality often associated with palatable food (Maehashi et al. 1999). This is primarily due to glutamate, an excitatory amino acid that activates the umami taste receptor complex (Yin Zhang et al. 2017). Certain peptides formed during hydrolysis also contribute to savory or meaty flavor notes, which have been reported in several protein hydrolysates (Schlichtherle‐Cerny and Amadò 2002; Fuke and Shimizu 1993; Zheng et al. 2023). The savory sensation can be further enhanced by the addition of nucleotides, such as inosine monophosphate, or by certain salts, both of which act synergistically with glutamate to intensify umami perception (Yin Zhang et al. 2017). Beyond its sensory role, glutamate also serves as a metabolic intermediate in the tricarboxylic acid cycle and functions as a neurotransmitter linking its flavor contribution to broader biochemical relevance (Peng et al. 1993). In contrast to these desirable taste contributors, bitterness is the most frequently reported negative taste attribute in protein hydrolysates.

Bitterness in protein hydrolysates is often attributed to low‐molecular‐weight peptides, typically below 1 kDa, containing hydrophobic or aromatic amino acids such as phenylalanine, tryptophan, tyrosine, isoleucine, valine, proline, and histidine (Idowu et al. 2019; Aspevik et al. 2016b). The position of these amino acids at internal, mixed internal/terminal, and terminal locations in the peptide sequence influences the bitter taste in long‐, medium‐, and short‐chain peptides, respectively (Matoba and Hata 1972; Idowu and Benjakul 2019). The bitter taste arises from the interaction of hydrophobic peptides with the bitter taste receptors in the oral cavity (Idowu and Benjakul 2019; Ishibashi et al. 1988). Several attempts have been made to predict bitterness in protein hydrolysates from peptide features, most notably the Q‐rule (Ney 1971), which estimates bitterness from the ratio of hydrophobic surface area to molecular weight. However, these quantification methods have shown limited accuracy and weak correlations with sensory perception (Toelstede and Hofmann 2008). Therefore, bitterness is currently best assessed by trained sensory panels (Toelstede and Hofmann 2008).

In addition to peptides, certain non‐ to low‐volatile biogenic amine compounds can also affect the sensory properties of protein hydrolysates. These include cadaverine, putrescine, β‐phenylethylamine, tyramine, and histamine, which are produced via enzymatic decarboxylation of amino acids and may arise naturally in the side‐stream materials or through microbial activity (Schirone et al. 2022). These compounds, typically detected via liquid chromatography, contribute to off‐flavors as well as fishy, stale, and decaying flesh‐like odors in protein hydrolysates and, when present in excessive amounts, may pose adverse health effects (Schirone et al. 2022; Özogul and Fatih 2019).

### 3.2 Volatile Compounds: Aromas and Odors

Volatile compounds strongly influence the aroma and odor characteristics of protein hydrolysates, contributing to both desirable and undesirable notes. Although volatile compounds function primarily via odor, many are perceived as part of the overall flavor and are often described using taste‐like terms (e.g., waxy or metallic) (Calkins and Hodgen 2007; Newman et al. 2014). One of the main metabolic processes that leads to the formation of volatile compounds is non‐microbial lipid oxidation (Domínguez et al. 2019). Lipid oxidation may occur both in the raw materials prior to hydrolysis and during processing, as heating, pH changes, and tissue disruption can accelerate oxidative reactions (Domínguez et al. 2019; Yarnpakdee et al. 2012c). This occurs due to autoxidation, photo‐oxidation, or enzyme‐catalyzed oxidation, producing primary lipid oxidation products such as lipid hydroperoxides, and secondary products including aldehydes, ketones, alcohols, and hydrocarbons (Domínguez et al. 2019). Among these, aldehydes are the major contributors to unpleasant odors, with compounds such as hexanal, nonanal, malondialdehyde, hexanol, 2‐heptenal, and 1‐octanol that can impart grassy, waxy, rancid, metallic, fishy, and burnt sensory notes to the protein hydrolysates if not adequately controlled or removed (Calkins and Hodgen 2007; Domínguez et al. 2019; Hülsebusch et al. 2025).

Side streams rich in polyunsaturated fatty acids (PUFAs) are particularly prone to lipid oxidation, which often results in fishy odors and bitter tastes. For example, in soy and pea proteins, oxidation of unsaturated fatty acids, notably linoleic/linolenic (PUFAs) and oleic (a monounsaturated fatty acid), leads to the formation of hexanal, 2,4‐decadienal, nonanal, 1‐octanol, and other off‐flavor compounds. These volatile oxidation products contribute to grassy flavor, plastic‐like, oily/aldehydic, and various pungent sensory notes, respectively (Damodaran and Arora 2013; Zhang et al. 2020). Animal‐based side streams with high PUFA contents, especially from fish and meat, are among the most challenging to protect from lipid oxidation (Domínguez et al. 2019; Nikoo et al. 2022). Viscera are particularly prone to oxidative deterioration due to their high enzymatic activity and abundance of pro‐oxidants such as hemoglobin, myoglobin, and iron compounds. These factors accelerate autolysis and lipid oxidation, leading to rancid odors. Fish frames and heads, rich in phospholipids and PUFAs, are highly susceptible to oxidative degradation, while trimmings exhibit variable composition and may contain residual blood and connective tissue that introduce additional pro‐oxidants (Nikoo et al. 2022).

In grain‐fed beef, oxidation of linolenic fatty acids produces aldehydes such as hexanal, 2‐heptenal, and 2,4‐decadienal, which contribute to fatty and soapy flavors. In addition, oxidation of medium‐ and long‐chain fatty acids contributes to liver‐like off‐flavor or metallic taste (Calkins and Hodgen 2007). In fish, oxidation of eicosapentaenoic acid and docosahexaenoic acid forms hexanal, 2,4‐heptadienal, 2,6‐nonadienal, 1‐penten‐3‐one, and 1‐octene‐3‐ol, compounds that contribute to fishy odors (Sullivan Ritter and Budge 2012; Liu et al. 2024; Peinado et al. 2016; Lee et al. 2003). Even when lean muscle is used, or lipid‐rich fractions are removed, polar lipids such as phospholipids and free fatty acids, which are bound to proteins, may end up in the water‐soluble protein hydrolysates (Liang and Hultin 2005).

In some cases, free amino acids in protein hydrolysates can be metabolized into odor‐active volatile compounds through microbial catabolism and chemical pathways. Changes in temperature, pH, and storage duration strongly influence these pathways (Duong et al. 2019; Cheng et al. 2024). During microbial or chemical degradation, amino acids and other intermediates may undergo deamination, decarboxylation, or transamination, producing keto acids and other metabolites that can be further oxidized into volatile fatty acids, thereby affecting the odor of protein hydrolysates (Yaqi Wang et al. 2021; Al‐Kwradi and Altarawneh 2024; Gao et al. 2022). Protein fermentation by anaerobic microbes such as lactic acid bacteria may also produce a variety of odor‐active compounds (Yaqi Wang et al. 2021; Gao et al. 2022). Further Maillard reaction, a non‐enzymatic reaction that occurs between amino groups of protein, peptides, or amino acids with reducing sugars, can produce several volatile compounds, including pyrazines such as dimethylpyrazines and trimethylpyrazines (Yu Fu et al. 2020). These compounds contribute to browning through melanoidin formation and impart roasted nut, smoky, and sweet caramel‐like aromas (Yu Fu et al. 2020; Arsa and Theerakulkait 2018). Additionally, carbonyl groups of Maillard intermediates or lipid oxidation products can react with amino acids via Strecker degradation, generating odor‐active aldehydes: for example, phenylalanine into phenylacetaldehyde (honey‐like aroma) and leucine into 3‐methylbutanol (malty) (Gao et al. 2022; Meng et al. 2021). Further, sulfur‐containing amino acids such as cysteine and methionine undergo enzymatic or microbial degradation into sulfides, dimethyl sulfides, and methanethiol. These compounds are associated with off‐odors resembling rotten egg, cabbage, and decaying vegetable, respectively (Varlet and Fernandez 2010).

A well‐known nitrogen compound contributing to the fish‐like odor in marine‐sourced protein hydrolysates is the volatile biogenic amine trimethylamine (TMA) (De Vooys 2002; Lidbury et al. 2014). TMA primarily develops postmortem in fish and seafood through microbial reduction of the natural osmolyte trimethylamine N‐oxide (TMAO), which stabilizes proteins under high osmotic pressure (Wu and Bechtel 2008). TMA has an extremely low odor threshold, down to 0.00021 ppm (Leonardos et al. 1969). TMA levels in the raw material vary depending on species, tissue, and processing, and are particularly abundant in species that inhabit the deep ocean (Shumilina et al. 2016). Species such as cod, haddock, and shark contain high TMAO levels, explaining their strong odor potential (Fuentes‐Amaya et al. 2016; Yancey et al. 2014).

Various analytical techniques can be applied to detect volatile compounds (Barriuso et al. 2013), including UV–vis spectrophotometry, headspace solid‐phase microextraction, or dynamic headspace combined with gas chromatography and mass spectrometry (Domínguez et al. 2019) or ion mobility spectrometry (Wenjia Cui et al. 2023). These compounds can further be correlated with the sensory data from trained panelists and supported by statistical analysis to evaluate their aromatic attributes, intensities, and overall contribution to the sensomic profile (Daher et al. 2020; Cardinal et al. 2020). Methods can also be linked; for example, gas chromatography–olfactometry couples chemical separation with human sensory evaluation to directly identify and characterize odor‐active compounds (Mahmoud and Zhang 2024)

## 4 Processing Steps That Affect the Sensory Attributes of Protein Hydrolysates

Comprehending how processing influences the sensory characteristics of protein hydrolysates is essential to avoid detrimental effects. The sensory properties often result from a combination of inherent attributes in the side streams, defined by their composition and freshness, and those developed during processing, including enzymatic hydrolysis conditions, downstream processing, and microbial activity during storage. These influences can be broadly grouped into three processing stages: pre‐processing, peri‐processing, and post‐processing.

### 4.1 Pre‐Processing: Raw Material Composition and Freshness

The intrinsic composition of side streams used for enzymatic hydrolysis, particularly their lipid content and fatty acid profile, plays a fundamental role in determining the sensory quality of the resulting protein hydrolysates. Equally important is the freshness of the side streams, as it directly influences process yield, nutritional quality, and sensory properties (Vang et al. 2020). As discussed previously, oxidation of polyunsaturated and related lipids generates volatile compounds such as aldehydes and ketones that contribute to off‐odors and off‐flavors. Temperature fluctuations during storage can exacerbate this issue: Slow freezing promotes extracellular ice formation, disrupting cells and releasing pro‐oxidants, while slow thawing activates phospholipases and neutral lipases, increasing the content of free fatty acids (Flores et al. 2009; Thanonkaew et al. 2006). Moreover, prolonged storage intensifies undesirable sensory attributes; for example, lipid oxidation in marinated beef during the first 2 months of storage results in a significant increase in malondialdehyde, a key aldehyde linked to rancid/metallic notes (Al‐Dalali et al. 2022). In Atlantic cod, storage at 2°C for 15 days developed higher TMA levels than farmed cod or Atlantic herring, reflecting species‐specific differences in TMAO content that influence the strength of fishy odor (Özogul and Fatih 2019; Herland et al. 2009).

A satisfactory result is typically achieved by using fresh raw material and removing fractions and components responsible for off‐flavors or odors through washing, discarding, heating, and acid or alkali treatments (Leonard et al. 2023; Tan et al. 2023; Yarnpakdee et al. 2012b, Yarnpakdee et al. 2012a).

### 4.2 Peri‐Processing: Hydrolysis Process and Parameters

Proteases cleave peptide bonds to form peptides, with the extent of cleavage reported as the degree of hydrolysis (DH). In practice, protein‐rich side streams are hydrolyzed by one of three approaches: using endogenous enzymes present in the side stream, adding commercial enzymes, or employing a combination of both, to achieve the desired yield or product characteristics (Kristinsson and Rasco 2000; Clemente 2000; Nikoo et al. 2022; Czelej et al. 2022). The choice of enzyme and its dosage, along with process parameters such as time, temperature, and pH, influence the peptide profiles produced. Since certain peptides are bitter, the peptide profile produced during hydrolysis directly impacts sensory characteristics, making enzyme selection and operating conditions decisive for the final sensory quality.

Alcalase is one enzyme known to cause bitterness in the protein hydrolysates due to its catalytic specificity and its tendency to produce higher DH compared to other proteases such as Protamex, Flavourzyme, papain, bromelain, trypsin, and α‐chymotrypsin (Qiang Cui et al. 2021; Humiski and Aluko 2007; Idowu et al. 2019b; Xiaorui Sun et al. 2022). This effect is well illustrated in salmon protein hydrolysates, where bitterness intensity produced with Alcalase was significantly higher than that of hydrolysates generated using Promod671L and FoodPro PNL (formerly known as Protex7L), even though the DH values were comparable (Aspevik et al. 2016b). Bitterness often rises within a DH range of 4% to 40% (Rustad and Hayes 2012), although enzyme specificity can sometimes have a stronger impact than DH alone (Aspevik et al. 2016b). Beyond enzyme specificity and DH, process temperature can also shape sensory outcomes. Extended hydrolysis at temperatures above 50°C may promote Maillard reactions, as more free amino acids become available to interact with sugars in some feedstocks (Oh et al. 2013), potentially creating savory and umami compounds that can help mask bitterness.

### 4.3 Post‐Processing: Separation, Concentration, and Drying

During post‐processing, the soluble protein hydrolysate fraction is separated from oil and the insoluble fraction through centrifugation and filtration (Hou et al. 2017; Kristinsson and Rasco 2000). Different pore size membranes can be used to separate protein hydrolysates from bitter‐tasting peptides, salts, and other fine particles (Castro‐Muñoz et al. 2020). To preserve quality and ensure the stability of protein hydrolysates, further post‐processing steps such as concentration and drying are carried out. Removing water from protein hydrolysates is essential to prevent microbial growth, which can otherwise lead to spoilage, off‐flavors, and potential health risks (Stawick and Kornacki 2014; Dave and Routray 2018). Spray drying at temperatures higher than 100°C may accelerate the Maillard reaction in carp skin hydrolysates (Ye Dong et al. 2022). Freeze drying performed at temperatures below −20°C may result in residual moisture content trapped in gels or sticky masses if not done carefully (Ezhilarasi et al. 2013).

Additional processing steps, such as blending and formulation, can be explored to modify sensory attributes. For example, hydrolysates from different sources can be blended to standardize peptide composition or improve taste, and formulation involves adding flavors, stabilizers, or sweeteners to tailor the product for specific applications (Honrado et al. 2023; Ardila et al. 2025).

## 5 Strategies to Remediate Undesirable Sensory Properties in Protein Hydrolysates

**SCHEME 1:**
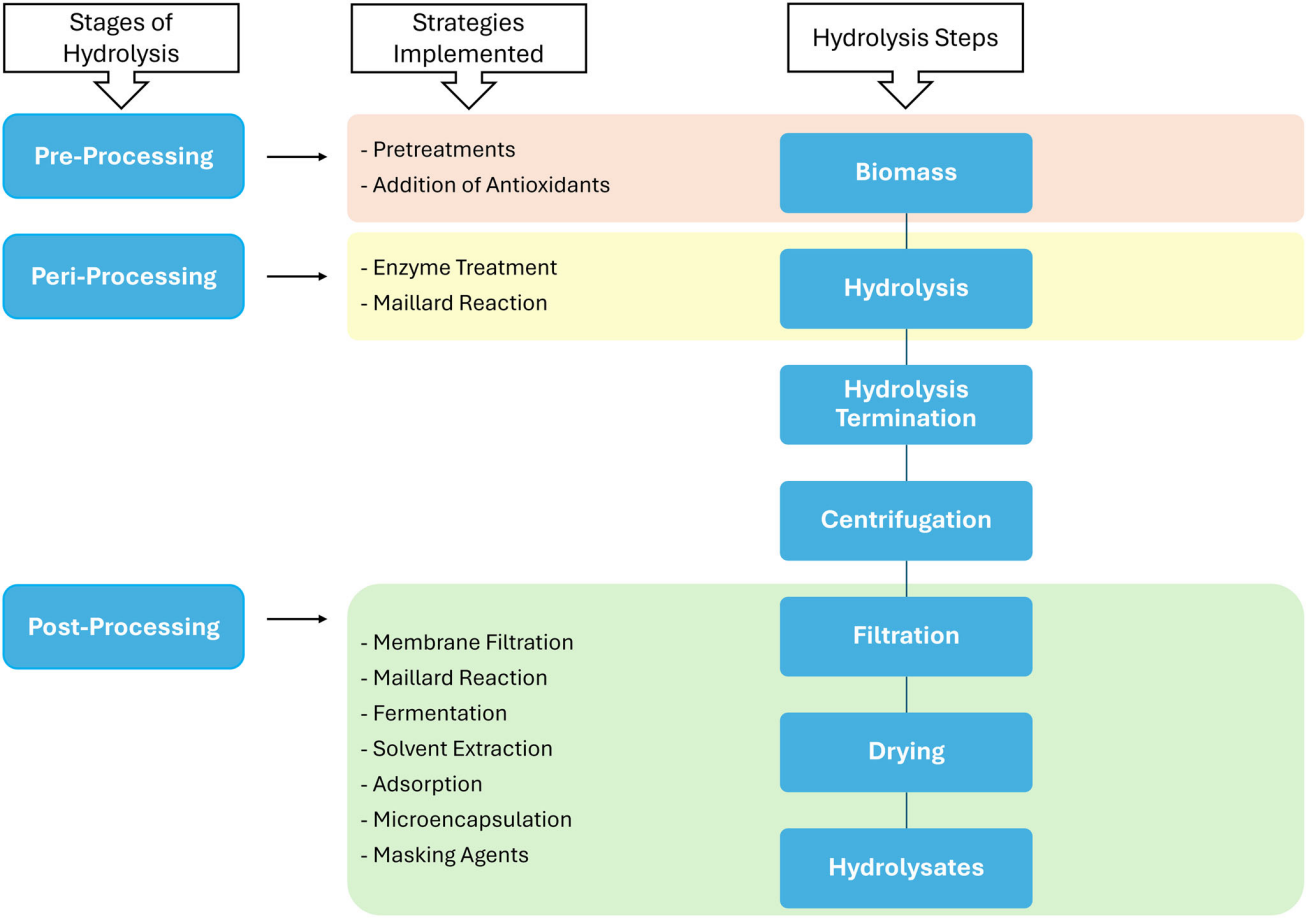
Strategies implemented at different stages of the hydrolysis process to improve the sensory properties in protein hydrolysates. The scheme is constructed based on insights gathered from multiple studies addressing protein hydrolysis and strategies aimed at mitigating bitter taste and off‐flavors (FitzGerald and O'Cuinn 2006; Y. Fu et al. 2019; Dai et al. 2024).

Strategies applied to remediate sensory attributes in protein hydrolysates should meet several criteria, such as not adversely affecting nutritional quality, meeting the ISO 22000 food safety standards for consumption (Panghal et al. 2018), being feasible for large‐scale or industrial application, and being both time‐ and cost‐efficient. Based on literature searches, we have identified 10 key strategies that have been reported to effectively reduce unwanted sensory properties in protein hydrolysates: pretreatment of side streams, application of antioxidants, enzymatic modification methods, use of Maillard reaction, fermentation processing techniques, solvent‐assisted extraction, adsorption removal techniques, membrane filtration processes, microencapsulation technology, and use of masking agents (Yarnpakdee et al. 2012a; Steinsholm et al. 2021; FitzGerald and O'Cuinn 2006; Yang et al. 2012; Halldorsdottir et al. 2013; Jacobsen et al. 2018; Shiguo Liu et al. 2022; Dauksas et al. 2004; Song et al. 2023; Newman et al. 2015; Synowiecki et al. 1996; Cheison et al. 2007). These strategies are implemented at different stages of the protein hydrolysis process, as illustrated in Scheme 1. Each strategy presents distinct advantages and disadvantages, including its compatibility with industrial settings, which are discussed below and summarized in Table 1.

**TABLE 1:** Strategies to modify sensory properties during protein hydrolysis: Its effect, processing stages, and industrial assessment criteria.

	Strategies	Effect on target (additive, neutral, and loss)	Processing stage (pre, peri, and post)	Assessment criteria					Ref.
				Targeted (T)/unspecific (U)	Food compliance	Extended time	Added cost	Industrial compatibility	
1	Pre‐treatment	Loss, neutral	Pre	T	✓	✓	✓	✓	^a*^
2	Antioxidants	Additive	Pre/Post	T	✓	✓	✓	✓	^b*^
3	Enzyme treatment	Neutral (processing aid)	Peri	T/U	✓	✓	✓	✓	^c*^
4	Maillard reaction	Neutral	Peri/Post	U	—	—	✓	✓	^d*^
5	Fermentation	Neutral (processing aid)	Post	T	✓	✓	✓	—	^e*^
6	Organic solvent–assisted extraction	Loss	Post	U	—	✓	✓	—	^f*^
7	Adsorption	Loss	Post	U	—	✓	✓	✓	^g*^
8	Membrane filtration	Loss	Post	T	✓	✓	✓	✓	^h*^
9	Micro‐encapsulation	Neutral	Post	U	✓	—	✓	✓	^i*^
10	Masking agents	Additive	Post	U	✓	—	✓	✓	^j*^

a*: Yarnpakdee et al. (2012b); Seth and Nath (1988); Hemker et al. (2020); Yuanyuan Wang et al. (2022); Sarteshnizi et al. (2019); Leygonie et al. (2012); Yarnpakdee et al. (2012a).

b*: Sarteshnizi et al. (2019); Lorenzo et al. (2018); Soendjaja and Girard (2024); Sørensen et al. (2023); Haizhou Wu et al. (2021); Yarnpakdee et al. (2012c); Chen et al. (2016).

c*: Jing Fu et al. (2011); Lei et al. (2021); Rosas‐Romero and Baratta (1987); Sharma et al. (2023); Goris et al. (2023).

d*: Yongsheng Zhang et al. (2019); Aljahdali and Carbonero (2019); Lineback et al. (2012); Poulsen et al. (2013).

e*: Meinlschmidt et al. (2016); Dzurendova et al. (2022); Smit et al. (2005); Vesković et al. (2022); Schlegel et al. (2021); Kieliszek et al. (2021).

f*: Sinthusamran et al. (2020); Lalasidis and Sjoberg (1978).

g*: Doulia et al. (2001); Jensen et al. (2011); Suh et al. (2000).

h*: Petrova et al. (2018); Steinsholm et al. (2021).

i*: Jacobsen et al. (2018); Sarabandi et al. (2018).

j*: Normah and Fasihah (2017); Qingliang Dong et al. (2017).

### 5.1 Strategy 1: Pretreatment of Side Streams to Reduce Off‐Flavors and Odors

Pretreatment of side streams prior to enzymatic hydrolysis may be optimized to promote desirable sensory attributes. For example, washing, lipid extraction, and alkaline solubilization in tilapia and mackerel mince removed phospholipids, myoglobulin, and heme/non‐heme iron before hydrolysis with Alcalase, resulting in improved oxidative stability (Yarnpakdee et al. 2012b, Yarnpakdee et al. 2012a). Similarly, removing the gall bladder, containing bile acid from cod viscera prior to hydrolysis, prevented bitter taste formation (Dauksas et al. 2004). For plant proteins, crushing, grinding, high‐pressure processing, ultrasonication, nitrogen flushing, and blanching were applied to extract lipids and inactivate the lipoxygenases and other endogenous enzymes to prevent the formation of off‐odor and off‐flavor compounds (Seth and Nath 1988; Hemker et al. 2020; Yuanyuan Wang et al. 2022; Sarteshnizi et al. 2019). As mentioned above, rapid freezing and thawing of by‐product materials can help prevent off‐flavors caused by cell damage and lipid oxidation (Leygonie et al. 2012).

### 5.2 Strategy 2: Use of Antioxidants to Prevent Oxidative Off‐Notes

Antioxidants are compounds that scavenge free radicals and protect cells from oxidative stress (Sarteshnizi et al. 2019; Elias et al. 2008). They can be incorporated during pre‐processing of raw material, enzymatic hydrolysis, and storage to prevent off‐odor and off‐flavor formation resulting from lipid oxidation and microbial activity (Lorenzo et al. 2018; Soendjaja and Girard 2024). However, the application of antioxidants specifically to enhance the sensory properties of protein hydrolysates remains underexplored in the literature. Nonetheless, recent studies have demonstrated the effectiveness of antioxidants in related contexts, such as preventing lipid oxidation in cod and herring side streams by dipping in rosemary extract and Duralox MANC (a mixture of rosemary extract, citric acid, ascorbic acid, and tocopherols) rich in antioxidants, respectively (Sørensen et al. 2023; Haizhou Wu et al. 2021). During peri‐processing of Nile tilapia whole fish mince, antioxidants such as EDTA and Trolox were added to suppress the formation of lipid pro‐oxidants, including both non‐heme and heme catalysts (Yarnpakdee et al. 2012c). Additionally, these antioxidants helped prevent the decomposition of lipid hydroperoxides, thereby reducing fishy odors and off‐flavors in protein hydrolysates. Research on the use of antioxidants for post‐processing of protein hydrolysates is limited. However, mixing tea polyphenols with clam protein hydrolysates has shown promising results in suppressing the formation of TMA (Chen et al. 2016). However, it did not effectively reduce the odor score from aldehydes and ketones compared to adsorption and masking treatment (Chen et al. 2016).

Many peptides generated by enzymatic hydrolysis also inherently possess antioxidant properties. These peptides can act as free radical scavengers or metal chelators, or regenerate existing antioxidants, offering a potentially intrinsic means of enhancing oxidative stability (Elias et al. 2008). To the best of our knowledge, leveraging these peptide‐derived antioxidant effects as a strategy to optimize sensory attributes has not yet been investigated.

### 5.3 Strategy 3: Enzymatic Approaches to Improve Sensory Properties of Protein Hydrolysates

As outlined above, the enzymatic hydrolysis process plays a pivotal role in shaping the sensory characteristics of protein hydrolysates, with enzyme choice, dosage, and hydrolysis conditions influencing the resulting peptide profiles and, consequently, their taste and aroma (Xiaoyu Liu et al. 2012; Eberhardt et al. 2021). Although several studies investigating DH do not report on sensory attributes, it is recognized that DH influences peptide size and can alter the sensory properties of protein hydrolysates (Saha and Hayashi 2001). High enzyme‐to‐substrate ratio increases the DH, protein solubility, and reduces peptide size, whereas an excessive substrate concentration can negatively affect these parameters (Chiodza and Goosen 2024; Silva et al. 2010). For example, hydrolyzing mussel meat protein using Protamex at an enzyme‐to‐substrate ratio exceeding 3% resulted in a high DH and generated umami flavor‐enhancing amino acids, such as glutamic acid and aspartic acid (Silva et al. 2010). Whey protein hydrolysates with a high initial total solids content exhibited less bitterness compared to those with low solids (Spellman et al. 2005). At low total solids, more hydrophobic peptides are available for enzymatic reaction, leading to a higher DH and increased formation of bitter peptides and amino acids. Hydrolyzing proteins at temperatures below 40°C may reduce overall sensory intensity but can also negatively impact the final protein yield (Palupi et al. 2010). Temperature, along with pH, significantly influences enzyme activity by affecting catalytic efficiency, reaction rate, and enzyme stability, all of which impact DH. For example, hydrolysis of wheat protein at pH 9 using Alcalase resulted in a higher DH compared to hydrolysis at neutral pH, primarily because Alcalase is more active under alkaline conditions (Kleekayai et al. 2022). Therefore, selecting an appropriate protease and optimizing the hydrolysis conditions are crucial to achieving optimal sensory attributes in protein hydrolysates.

The enzymes commonly used by industry, such as subtilisins, trypsins, pepsins, and papains, are all endoproteases (Yu Fu et al. 2019) that have broad specificity and cleave internal peptide bonds within proteins, often leading to bitter‐tasting hydrolysates. To reduce bitterness, exopeptidases such as carboxy‐ or amino‐peptidases can be used in combination with endoproteases (Saha and Hayashi 2001). These exopeptidases act on the peptides generated by endoproteases, cleaving hydrophobic amino acids from the N‐ and C‐termini, thus improving flavor and reducing bitterness during hydrolysis. However, the effectiveness of this approach depends on the protein's sequence and structure, as these factors influence the accessibility of hydrophobic residues and the formation of bitter peptides (Raksakulthai and Haard 2003). For example, bitterness in soy protein hydrolysates was reduced using experimental aminopeptidase and carboxypeptidase enzymes (Tchorbanov et al. 2011; Jing Fu et al. 2011). Commercial proteases like Flavourzyme, which contains both endo‐ and exopeptidases, have been shown to be effective in reducing bitterness in protein hydrolysates from pumpkin seeds and wheat gluten (Lei et al. 2021; Schlichtherle‐Cerny and Amadò 2002). However, Flavourzyme alone showed little effect on bitterness reduction in salmon protein hydrolysates (Dauksas et al. 2004; Sinthusamran et al. 2020). Interestingly, when Flavourzyme was combined with Protex 7L during hydrolysis, a slight reduction in bitterness was observed in salmon protein hydrolysates (Aspevik 2016a), despite this combination increasing the release of hydrophobic bitter amino acids (Aspevik 2016a).

Certain hydrolysis conditions involving proteases can promote the formation of polypeptide aggregates, plastein, created through enzymatic cross‐linking of peptides, which may potentially alter their functional and sensory properties (Synowiecki et al. 1996; Watanabe and Arai 1992). Plasteins are gel‐like, elastic, and poorly water‐soluble peptide aggregates with higher molecular weights (around 3 kDa) formed through condensation, transpeptidation, and/or non‐covalent interactions such as hydrophobic forces, hydrogen bonding, and electrostatic interactions (Gong et al. 2015; Li et al. 2020; Song et al. 2023). In cassava leaf hydrolysates, the plastein reaction catalyzed by pepsin significantly reduced bitterness compared to the original concentrates (Rosas‐Romero and Baratta 1987). Similarly, salmon protein hydrolysates were debittered using papain‐induced plastein reaction (Sharma et al. 2023). This method has also been used to reduce bitterness in protein hydrolysates from soybean, casein, chlorella, gluten, cod fish, yeast, and bovine hemoglobin using various commercial proteases (Synowiecki et al. 1996; Fujimaki et al. 1970). Plasteins require repeated washing steps to improve solubility, which limits their applications (Gong et al. 2015). Studies have also reported that bitterness may return if plasteins undergo enzymatic degradation (Yamashita et al. 1970). Moreover, downstream processing steps used to stabilize the product, reduce microbial loads, and control particle size, such as pasteurization, concentration, filtration, homogenization, and spray drying (Petrova et al. 2018), often involve high temperatures and pressures that can inhibit plastein formation or disrupt aggregates, thereby limiting the potential of this strategy in an industrial setting.

Recently, an enzyme‐driven strategy using a bacterial flavin‐containing monooxygenase was developed to convert the malodorous, fishy‐smelling TMA in salmon protein hydrolysates into odorless TMAO (Goris et al. 2020). This study was followed up with engineered thermostable variants of the enzyme that demonstrated significant reduction of TMA levels at industry‐relevant temperatures (Goris et al. 2023; Ree et al. 2026). This approach requires supplementation with the costly NADPH cofactor, necessitating process optimization to improve its feasibility for industrial‐scale application.

### 5.4 Strategy 4: Application of Maillard Reaction for Flavor Enhancement and Bitterness Masking

The Maillard reaction mentioned earlier can be strategically exploited for flavor enhancement in protein hydrolysate products. The reaction typically begins at moderate temperature (50–70°C), with its rate increasing at temperatures above 100°C (Rauh and Xiao 2022). Notably, thermal inactivation of enzymes can promote the formation of Maillard reaction products (Jianbin Liu et al. 2015). At lower temperatures (<50°C), the reaction may still occur, but proceeds more slowly (Oh et al. 2013; Rauh and Xiao 2022). Browning develops more rapidly under alkaline conditions than acidic ones (Arsa and Theerakulkait 2018). The specific types of amino acids and reducing sugars present, along with reaction parameters such as temperature, time, and pH, play crucial roles in flavor development during the Maillard reaction (Yu Fu et al. 2020; Liu et al. 2022). The Maillard reactions may as well reduce the nutritional value of protein hydrolysates by destroying amino acids, particularly the essential lysine, and/or by inducing cross‐linking between protein chains (Aljahdali and Carbonero 2019). Additionally, the brown pigments formed during the reaction may pose toxicity concerns, as they are commonly associated with compounds such as acrylamide and advanced glycation end products (Lineback et al. 2012; Poulsen et al. 2013).

Recently, the Maillard reaction coupled with sugar‐derived natural deep eutectic solvents (NADES) was used to enhance umami flavor and reduce the off‐flavors in pea protein hydrolysate (Huayang Wang et al. 2024). This approach enabled the reaction to proceed under notably milder conditions (55°C for 90 min) compared to traditional methods. The method is relatively cost‐effective due to lower heating requirements and is considered safe for industrial applications (Huayang Wang et al. 2024; Alsaidi and Thiemann 2025). However, the study did not assess the economic feasibility of scaling up NADES use, nor did it evaluate the efficiency and recycling potential of NADES to minimize waste at an industrial scale.

### 5.5 Strategy 5: Fermentation for Improvement of Sensory Attributes

Fermentation is a sustainable, cost‐effective method to improve the sensory qualities of protein hydrolysates using bacterial (e.g., lactic acid bacteria, *Bacillus* spp., *Saccharomyces cerevisiae*) and fungal species (e.g., *Saccharomyces cerevisiae*, *Candida* spp., *Aspergillus* spp., and *Rhizopus* spp.) (Meinlschmidt et al. 2016; Dzurendova et al. 2022). Microbes employ various mechanisms to enhance sensory properties in protein hydrolysates, including enzymatic degradation of bitter peptides by proteolytic enzymes such as peptidases and carboxy‐ and aminopeptidases. They also convert bitter and off‐flavor compounds into less objectionable metabolites through metabolic transformations and modulate microbial communities to suppress spoilage microorganisms while promoting flavor development (Kieliszek et al. 2021; Smit et al. 2005; Vesković et al. 2022). For example, *Lactobacillus perolens, Actinomucor elegans*, and *Rhizopus oryzae* have been shown to reduce bitterness and beany flavor in soy protein hydrolysates (Meinlschmidt et al. 2016). These microbes produce carboxy‐ and aminopeptidases that selectively cleave hydrophobic amino acids at C‐ and N‐termini of peptides. Similarly, lactic acid bacteria have been shown to reduce bitter taste and modify the taste of lupin hydrolysates, imparting cocoa and malty flavor notes (Schlegel et al. 2021). However, neither of the above‐mentioned microbes was able to completely debitter the protein hydrolysates. Fermentation is a complex process that requires the selection of microbes specific to the targeted protein hydrolysates to effectively improve their sensory properties. Often, sensory changes induced by microbial activity during fermentation are observed only after extended periods, typically 6 to 24 h (Schlegel et al. 2021; Meinlschmidt et al. 2016). This prolonged duration can significantly increase the time, energy, and cost required to complete the process.

### 5.6 Strategy 6: Organic Solvent‐Assisted Extraction for Removal of Bitter Compounds

Solvents can be used to selectively extract bitter‐tasting compounds from protein hydrolysates. This technique involves creating a two‐phase system consisting of an organic phase and an aqueous phase. When the solvent is mixed with water‐solubilized protein hydrolysates, hydrophobic peptides preferentially partition into the organic phase via hydrophobic interaction, while non‐bitter peptides and other components remain in the aqueous phase (Liu et al. 2022). Alcohols such as azeotropic secondary butyl alcohol, 2‐butanol, and isopropanol have been used to remove bitterness from soy, cod, herring, and salmon protein hydrolysates (Sinthusamran et al. 2020; Lalasidis and Sjoberg 1978). Further steps, such as stirring hydrolysates with alcohol followed by centrifugation, have been applied to aggregate hydrophobic compounds. This process enhances the interaction between hydrophobic compounds and the hydrophobic domains of the solvent, improving their solubilization. In one study, butanol demonstrated a higher extraction yield of bitter peptides from salmon protein hydrolysates compared to isopropanol (Sinthusamran et al. 2020).

Solvent‐based extraction requires a significant amount of solvents for industrial production, which increases the cost. The isolated bitter peptides often require additional processing steps to improve taste, aroma, and overall quality before incorporation into food products (Lalasidis and Sjoberg 1978; Singh et al. 2020; Saha and Hayashi 2001), which further adds to the expenses. Although preliminary studies have shown that secondary butyl alcohol exhibits low toxicity in rat models (Lalasidis and Sjoberg 1978), it remains essential to thoroughly investigate the safety of all solvents before their use in food formulations.

### 5.7 Strategy 7: Adsorption Techniques to Selectively Remove off‐Flavor Molecules

Using adsorption resins prior to solvent extraction can further improve the sensory attributes of protein hydrolysates, as these resins have hydrophobic cavities that trap bitter peptides. Macroporous adsorption resins have been used prior to alcohol washing to attenuate undesired sensory attributes in protein hydrolysates from fish skin and foxtail millet (Wasswa et al. 2007; Kamara et al. 2011). Likewise, cholestyramine resins were used before butanol treatment to improve the taste and odor of hydrolysates from cod by‐products (Dauksas et al. 2004). Although bitterness and malodor were effectively removed, solvents negatively affected the taste and protein yield (Dauksas et al. 2004).

Debittering and purification of protein hydrolysates can also be performed by selective adsorption without prior treatment with an organic solvent (Liu et al. 2022). Activated carbons, polymeric resins, and siloxanes can absorb and thereby remove aromatic and hydrophobic compounds from the hydrolysates. This effectively reduces the levels of bitter‐tasting hydrophobic peptides and amino acids. Aromatic amino acids such as tyrosine, tryptophan, and phenylalanine are particularly susceptible to adsorption onto these materials, primarily due to short‐range Van der Waals interactions (Doulia et al. 2001; Jensen et al. 2011). Several studies have reported a reduction in bitter taste through adsorption, but high doses of adsorbent materials and long reaction times may significantly increase processing costs (Cheison et al. 2007; Suh et al. 2000). Furthermore, many of the hydrophobic amino acids targeted for adsorption are essential for human nutrition, and their removal could reduce the nutritional value of hydrolysates.

### 5.8 Strategy 8: Membrane Filtration for Fractionation and Purification of Hydrolysates

After the separation of solids and fat during post‐processing, further refinement is typically necessary to produce a palatable product. This includes the removal of fine particles, traces of fat, salt, bitter‐tasting peptides, and small molecular volatiles that may cause stale or unpleasant flavors. One effective method for achieving such refinement is membrane filtration (Castro‐Muñoz et al. 2020), which enables fractionation of compounds and can be classified into microfiltration, ultrafiltration, and nanofiltration based on the molecular size of particles removed and retained in the products. Microfiltration, with pore sizes ranging from 0.1 to 10 µm, is effective for removing lipids and suspended particles. Ultrafiltration (pore size: 1–100 nm), operating within a molecular weight range of approximately 0.3 to 1000 kDa, is used to fractionate products based on peptide size distributions. Nanofiltration (pore size: 0.5 to 2 nm), targeting molecules between 0.2 and 2 kDa, can retain peptides while allowing smaller molecules to pass through (Castro‐Muñoz et al. 2020). It also removes water from hydrolysates, concentrating the products and potentially reducing unfavorable sensory attributes (Petrova et al. 2018). Nanofiltration of fish protein hydrolysates has been shown to significantly reduce the concentration of small flavor‐contributing metabolites, such as TMA, resulting in a lower intensity of sensory attributes (Steinsholm et al. 2021). The perception of bitterness, however, increased, likely due to the retention of peptides known to contribute to bitter taste.

### 5.9 Strategy 9: Microencapsulation to Mask Undesirable Flavors and Protect Peptides

Microencapsulation is a widely used strategy to improve the sensory properties of protein hydrolysates. This technique involves coating hydrolysate particles with a protective layer that helps prevent the release of undesirable flavors and aromas. Several methods can achieve microencapsulation, including spray‐drying, freeze‐drying, coacervation, electrospraying, and extrusion (Gouin 2004). It has been successfully applied to casein, whey, and chicken protein hydrolysates to optimize sensory attributes (Yang et al. 2012; Kurozawa et al. 2009; Sarabandi et al. 2018). Among all the methods of microencapsulation, spray‐drying is the most used (Sarabandi et al. 2018), where protein hydrolysates are atomized into a hot drying gas. This process rapidly dries the droplets, forming microcapsules. The protective coating can be made from a variety of materials such as carbohydrates, lipids, and proteins (Sarabandi et al. 2018). Maltodextrin has been used to microencapsulate casein hydrolysates and has been shown to reduce the bitterness of pastilles enriched with encapsulated hydrolysates compared with both non‐hydrolyzed casein and non‐encapsulated casein hydrolysates (Sarabandi et al. 2018). In addition, microencapsulation did not affect the antioxidant properties of the casein hydrolysates (Sarabandi et al. 2018), suggesting that the technique may help protect peptides from lipid and protein oxidation, although this protective effect was not explicitly stated in the paper. Additional research is required to verify the impact of microencapsulation on lipid and protein oxidation in protein hydrolysates. One major drawback of this technique is that it requires very high heating (>120°C), which may not be economical at an industrial scale and may result in unwanted Maillard reaction products in hydrolysates (Yang et al. 2012; Kurozawa et al. 2009; Jacobsen et al. 2018).

Recently, new microencapsulation techniques such as electrospraying and electrospinning have gained attention for encapsulating bioactive peptides without the use of heat. These methods have been successfully applied to protect bioactive compounds like omega‐3 PUFAs in fish oil from oxidation and may be more cost‐effective than spray‐drying (Jacobsen et al. 2018). In the future, these techniques could be explored for microencapsulating protein hydrolysates to evaluate their impact on sensory attributes.

### 5.10 Strategy 10: Use of Masking Agents to Suppress Unpleasant Taste and Odors

Masking agents are substances used to suppress undesirable taste and odor in protein hydrolysates by introducing new flavors and aromas, rather than eliminating the original sensory properties. The taste and odor of protein hydrolysates can be improved by adding sweeteners, flavors, and salt, which may be natural or synthetic. For example, bitterness in sodium caseinate hydrolysate was effectively masked using sucralose. The bitter intensity was further reduced in its model beverage by adding vanilla flavor (Newman et al. 2015). This strategy aligns well with consumer preferences, as validated by sensory panelists and electronic tongue analysis. Similarly, natural sweetness from β‐cyclodextrin was used to debitter protein hydrolysates derived from clam (Normah and Fasihah 2017). Moreover, an inclusion complex of neohesperidin dihydrochalcone with glucosyl‐β‐cyclodextrin was more effective in masking bitterness in corn peptide hydrolysate than the individual sweeteners used separately (Qingliang Dong et al. 2017). Sodium chloride has also been applied to mask bitterness in egg white and hen meat protein hydrolysates in a concentration‐dependent manner, leveraging its salting‐in effect (Qingbiao Xu et al. 2019a). This effect occurs as Na^+^ ions interact with positively charged hydrophobic peptides, inhibiting their binding to bitter taste receptors. In whey protein hydrolysates, salts (sodium acetate, monosodium glutamate, and sodium gluconate) and nucleotides reduced bitterness (Leksrisompong et al. 2012). However, this approach altered the overall taste profile by suppressing other desired flavors such as sweetness and sourness from vanilla, chocolate, and umami peptides.

In addition to sweeteners and salts, long‐chain fatty acids such as sodium stearate, palmitic acid, laurate, linolenic acid, and oleic acid have been explored to mask bitter‐tasting substances (Ogi et al. 2015). These compounds inhibit bitter taste receptors by forming an insoluble binary complex between fatty acids and bitter taste compounds, offering an alternative mechanism for taste improvement. Protein hydrolysates rich in glutamic acid, aspartic acid, and arginine can mask the bitter taste while increasing the umami flavor in bitter foods and other protein hydrolysates. For example, umami peptide‐rich protein hydrolysates derived from beef and hen raw materials were selectively produced using specific proteases to inhibit quinine bitterness by blocking bitter taste receptors (Qingbiao Xu et al. 2019b; Chunlei Zhang et al. 2018). Masking bitterness in protein hydrolysates presents several challenges, including the careful selection of masking agents and their optimal concentrations. The interactions between these agents and other compounds within the hydrolysates must be evaluated on a case‐by‐case basis, as improper assessment can alter the nutritional profile and potentially pose health risks, especially with the use of artificial sweeteners (Gauthier et al. 2024). Additionally, incorporating masking steps increases processing costs, which must be balanced against the benefits.

Taken together, the 10 strategies reviewed above address different sensory challenges with varying relevance for plant‐, animal‐, and marine‐derived protein hydrolysates. Because these challenges differ markedly between sources, we identified from the literature the strategies most applicable to each. Table 2 summarizes these source‐specific approaches by linking raw material, fraction, dominant sensory contributors, and effective mitigation methods, providing a clear overview of how remediation strategies diverge across protein origins.

**TABLE 2:** Summary of strategies applied to improve sensory properties in plant, animal, and marine protein hydrolysates.

Raw material	Fraction	Strategy	Implementation	Acts on sensory contributors	Sensory Improvement	Ref.
					Odor (O)/taste (T)	
**Marine protein**						
Nile tilapia *(Oreochromis niloticus)* and mackerel mince	Muscle	Pre‐treatment	Washing, lipid extraction via alkaline solubilization	Lipid oxidation	O	Yarnpakdee et al. 2012a, Yarnpakdee et al. 2012b)
Cod *(Gadus macrocephalus)* and Nile tilapia (*Oreochromis niloticus*)	Head, tail, and fins		Teeth and gills removed; followed by ultrasonication; high‐pressure enzyme hydrolysis/freezing	Unfolds protein aggregates, reduces bitterness, and increases odorless compounds	O/T	Hemker et al. (2020); Wang et al. (2022)
Cod (*Gadus morhua*)	Viscera		Gallbladder removal	Bitterness from bile acid	T	Dauksas et al. (2004)
All fish species	Whole fish		Lipid oxidation	High‐pressure‐assisted freezing and thawing	O	Leygonie et al. (2012)
Sind sardine (*Sardinella sindensis*)	Muscle		N_2_ flushing + pistachio green hull antioxidant	Lowers O_2_ level and reduces lipid oxidation	O	Sarteshnizi et al. (2019)
Cod *(Gadus morhua)*	Head, tail, fins, backbone, and intestine	Antioxidant	Rosemary extract	Lipid Oxidation	O	Sørensen et al. (2023)
Herring (*Clupea harengus*)			Duralox MANC			Wu et al. (2021)
Nile tilapia (*Oreochromis niloticus*)	Muscle		EDTA and tolox	Inhibits pro‐oxidants like heme and non‐heme	O/T	Yarnpakdee et al. (2012a)
Clam (*Paphia undulata*)	Muscle		Tea polyphenols	Reduces aldehydes and the formation of TMA	O	Chen et al. (2016)
Mussel (*Perna Perna*)	Muscle	Enzyme treatment	Protamex	Increase in glutamic and aspartic acid	T	Silva et al. (2010)
Salmon (*Salmo salar*)	Head and backbone		FoodPro PNL (Protex7L)/Protex671L	Less bitter peptides produced during hydrolysis	T	Aspevik et al. (2016b)
Salmon (*Salmo salar*)	Frames		Flavourzyme in combination with Protex 7L	Releases amino acids from bitter peptides at C‐/N‐terminals	T	Aspevik (2016a)
			Papain‐induced plastein reaction	Bitter peptides are converted into less‐bitter aggregates	T	Sharma et al. (2023)
			Engineered flavin–containing monooxygenase	Converts fishy odor TMA to odorless TMAO	O	Goris et al. 2020, Goris et al. 2023)
		Organic solvent–assisted extraction + masking agent	2‐butanol +β‐cyclodextrin	Removes bitter peptides	T	Sinthusamran et al. (2020); Lalasidis and Sjoberg (1978); Singh et al. (2020)
		Organic solvent–assisted extraction	2‐butanol			
Cod and herring	Cod deboned fillet and herring (whole)		Azeotropic secondary butyl alcohol			
Grass carp (*Ctenopharyngodon idella);* Nile perch (*Lates niloticus);* Nile tilapia (*Oreochromis niloticus*)	Fish skin	Adsorption	Macroporous resin	Bitter peptides and salts removed	O/T	Wasswa et al. (2007)
Salmon (*Salmo salar*) and Cod (*Gadus morhua*)	Heads and backbone	Membrane filtration	Nanofiltration	NF membrane removes TMA and other flavor‐enhancing metabolites	O	Steinsholm et al. (2021)
Angelwing clam (*Pholas orientalis)*	Muscle	Masking agent	β‐cyclodextrin	Suppresses bitter taste and flavors/aromas	T	Normah and Fasihah (2017)
Nile tilapia (*Oreochromis niloticus*)	Whole	Fermentation	Enzymatic degradation by *Hanseniaspora pseudoguilliermondii*	Reduces fishy odor and bitter taste	O/T	Shiguo Liu et al. (2022)
**Animal protein**						
Beef, pig, and poultry meat	Muscle	Pre‐treatment	High‐pressure thawing and freezing	Reduces lipid oxidation	O/T	Leygonie et al. (2012)
		Antioxidant	Berries and plant extracts	Reduces lipid and protein oxidation		Lorenzo et al. (2018)
Hen	Carcasses deboned and skinned	Enzyme treatment	Protex 6L and Protex 50 FP	Releasing fewer bitter peptides	T	Xu et al. (2019b)
Milk	Whey		High solid‐to‐liquid ratio	Reduces the degree of hydrolysis	T	Spellman et al. (2005)
Bovine	Blood	Papain‐induced plastein reaction with glutamic acid	To incorporate glutamic acid into plasteins	T	Synowiecki et al. (1996)
	Whey	Adsorption	Active carbon	Bitter peptides and salts removed	T	Cheison et al. (2007)
		Masking agents	Salts and nucleotides	Inhibited bitter taste	T	Leksrisompong et al. (2012)
	Sodium caseinate		Sucrose and vanilla flavoring/Na^+^ salts	Suppresses bitter taste and flavors/aromas	T	Newman et al. (2015)
	Whey	Micro‐encapsulation	Spray drying using maltodextrin/β‐cyclodextrin for encapsulation	Reduces bitterness and prevents the release of aromas	O/T	Yang et al. (2012); Sarabandi et al. (2018); Kurozawa et al. (2009)
Chicken	Breast meat					
		Maillard reaction	Reducing sugar reacts with the amino groups of peptides	Boosts umami and masks bitterness	T	Zhang et al. (2019)
	Egg white	Masking agents	Sodium chloride	Suppress bitterness by inhibiting the hydrophobic peptide interaction with bitter taste receptors	T	Xu et al. (2019a)
	Carcass					
**Plant protein**						
Soy	Cotyledons	Pre‐treatment	Crushing, grinding, and blanching	Inactivates lipoxygenases; reduces lipid oxidation	O/T	Seth and Nath (1988)
Pea and soy	Bean	Antioxidant	Polyphenols (e.g., grape seed extract, green tea extract, catechin, and tannic acid)	Reduces lipid oxidation	O/T	Soendjaja and Girard (2024)
Soy	Bean	Enzyme treatment	Carboxypeptidase from *Actinomucor elegans;*	Releases amino acids from bitter peptides at C‐/N‐terminals	T	Fu et al. (2011)
Pumpkin	Seeds		Aminopeptidases from *Bacillus axarquiensis*		T	Lei et al. (2021)
Wheat hydrolysates	Gluten		Flavourzyme and glutaminase	Releases amino acids from bitter peptides at C‐/N‐terminals + conversion of free glutamine into glutamic acid (umami flavor)	T	Schlichtherle‐Cerny and Amadò (2002)
Cassava hydrolysates	Leaf		Pepsin‐induced plastein reaction	Bitter peptides are converted into less‐bitter aggregates	T	Rosas‐Romero and Baratta (1987)
Pea hydrolysates	Bean	Maillard reaction, including NADES	Reducing sugar reacts with the amino groups of peptides	Boosts umami and masks bitterness	T	Wang et al. (2024)
Lupin/soy hydrolysates	Bean	Fermentation	Enzymatic degradation by LAB (*Bacillus* spp.) and fungi (*Saccharomyces cerevisiae*); suppression of off‐odor‐producing microbes	Reduces beany flavor and bitterness and adds cocoa and malty flavor	O/T	Meinlschmidt et al. (2016); Schlegel et al. (2021)
Foxtail millet	Seed	Adsorption	Macroporous resin	Bitter peptides and salts removed	T	Kamara et al. (2011); Suh et al. (2000)
Corn	Gluten		Active carbon			
		Masking agent	Inclusion complex of neohesperidin dihydrochalcone with glucosyl‐β‐cyclodextrin	Suppresses bitter taste and flavors/aromas	T	Dong et al. (2017)
Rice	Bran	Masking + micro‐encapsulation	Fructose added to hydrolysates and spray dried	Increase in Maillard products with cocoa‐, nut‐, and milk‐like odor	O/T	Arsa and Theerakulkait (2018)

## 6 Conclusion and Future Perspectives

A deeper understanding of the causes of various sensory attributes is critical for selecting targeted strategies to remediate undesirable characteristics. Most current approaches focus primarily on debittering peptides (Yu Fu et al. 2019; Hu et al. 2023; Idowu and Benjakul 2019a; Raksakulthai and Haard 2003), while odor‐specific solutions remain limited for both plant and animal proteins (Goris et al. 2020; Dai et al. 2024). At present, there is a lack of comprehensive studies on the factors that influence both taste and odor, and their combined impact on the sensory properties of protein hydrolysates. Nevertheless, since taste and odor are closely interconnected, we encourage future research to address both simultaneously to achieve more effective improvements in the overall sensory quality of protein hydrolysates.

Recent literature has also highlighted emerging technologies aimed at addressing unfavorable sensory attributes, including novel targeted enzyme‐based strategies, innovative heat‐free microencapsulation techniques, and Maillard reactions conducted in novel green solvents (Jacobsen et al. 2018; Goris et al. 2020; Huayang Wang et al. 2024). These three sub‐strategies are still in early development and require further optimization across a variety of protein hydrolysates to advance their technology readiness levels for industrial application.

This article identified and reviewed 10 strategies for improving sensory attributes, each tested in isolated studies on plant‐, animal‐, and marine‐derived protein hydrolysates. In plant‐derived hydrolysates, the most common off‐flavors are beany and grassy notes arising from lipid oxidation and lipoxygenase activity, while bitterness and astringency often originate from phenolic compounds (Damodaran and Arora 2013; Caimeng Zhang et al. 2020; Hülsebusch et al. 2025; Leonard et al. 2023; Soendjaja and Girard 2024). These issues can be mitigated through pre‐treatments such as blanching, high‐pressure processing, and ultrasonication, as well as through the use of antioxidants, enzymatic or fermentation‐based flavor modification, Maillard reactions, or masking agents (Seth and Nath 1988; Hemker et al. 2020; Yuanyuan Wang et al. 2022; Sarteshnizi et al. 2019; Elias et al. 2008; Soendjaja and Girard 2024). Animal‐derived hydrolysates typically develop metallic or liver‐like off‐flavor notes related to blood components and oxidation, which may also enhance bitterness (Domínguez et al. 2019; Calkins and Hodgen 2007). Strategies such as adding chelators or antioxidants during hydrolysis, applying the plastein reaction, using microencapsulation, or incorporating masking agents have shown potential for improving their sensory properties (Yang et al. 2012; Newman et al. 2015; Synowiecki et al. 1996; Cheison et al. 2007; Leygonie et al. 2012; Lorenzo et al. 2018; Spellman et al. 2005; Yongsheng Zhang et al. 2019). Marine‐derived hydrolysates remain the most challenging due to natural high content of TMAO and PUFAs in the raw materials which can lead to formation of TMA and secondary oxidation products that contribute to strong fishy and rancid odors (Liu et al. 2024; Yarnpakdee et al. 2012c). Maintaining raw material freshness, applying antioxidants, using appropriate pre‐treatments and membrane filtration, or enzymatically converting TMA to TMAO can help reduce these effects (Goris et al. 2020, Goris et al. 2023; Dauksas et al. 2004). While these mitigation strategies depend strongly on the specific raw material fraction, no comparative studies evaluating multiple approaches on the same biomass have been reported. Therefore, our assessment is based on the reported advantages and disadvantages of each method, acknowledging that the criteria used may not be equally weighted or relevant across all protein hydrolysate applications. Additionally, several strategies discussed are still at experimental or development stages, while others are likely available and applied at an industrial scale. It should also be noted that some strategies, for example, solvent‐assisted extraction, plastein formation, or extended fermentation, are unlikely to be practical for industrial production due to high cost, complexity, or incompatibility with workflows. Although the industrial perspective, like cost estimates and technology readiness levels, is rarely reported in scientific literature, such information is essential to determine industrial feasibility and scalability.

To fully confirm that a targeted sensory attribute has been reduced or eliminated, protein hydrolysates must be evaluated using complementary analytical assessments alongside sensory tests. Analytical techniques are sensitive to specific compounds but cannot capture the full complexity of the sensory experience. Additionally, factors such as the trained sensory panelists’ age, testing time, environment, and individual health should be carefully considered, as they can influence sensory evaluation outcomes. Given the complexity of taste and odor, removing one sensory attribute may reveal underlying attributes, meaning remediation strategies could potentially expose new undesirable notes. In some cases, remediation strategies may introduce new properties, including potentially toxic compounds. For example, Maillard reaction products formed at high temperatures can release acrylamide, hydroxymethylfurfural, and advanced glycation/lipoxidation compounds, which may pose health risks (Zha et al. 2015; Habinshuti et al. 2022; Ames 2009). Analyses should also confirm that implementing sensory remediation strategies does not adversely affect the overall product quality, including protein yield, nutritional value, and compliance with intended applications. For example, protein hydrolysates produced via enzymatic treatment and fermentation have been shown to maintain protein yield and nutritional quality while meeting ISO 22000 food safety standards for human consumption, although these processes can be costly and time‐consuming. Consequently, further investment in optimizing the cost and efficiency of these two strategies is recommended to develop products that satisfy consumer expectations.

In summary, enhancing sensory analysis of protein hydrolysates requires a holistic strategy, one that can address both taste and odor through rigorous analytical assessment, sensory evaluation, and statistical modeling to fully characterize its sensory quality. Promising strategies such as enzymatic treatment and fermentation need further research to optimize their cost effectiveness at an industrial scale, while emerging technologies need additional evaluation regarding their effectiveness, safety, and scalability. It is also vital to investigate the effect of different strategies on a standardized biomass to identify the most effective approach for producing high‐quality protein hydrolysates free from undesirable sensory attributes. Ultimately, integrating knowledge from sensory science, food toxicity, and process engineering will be key to developing innovative and sustainable production of protein hydrolysates that meet both industry standards and consumer preferences.

## Author Contributions

**Tejaswini R. B. Ramakrishna**: conceptualization; investigation; writing – original draft; writing – review and editing. **Tone Aspevik**: investigation, writing – review and editing. **Birthe Vang**: writing – review and editing, investigation. **Kjersti Lian**: investigation, writing – review and editing. **Charlotte Jacobsen**: investigation, writing – review and editing. **Volha Shapaval**: investigation, writing – review and editing. **Pål Puntervoll**: writing – review and editing, conceptualization. **Gro E. K. Bjerga**: conceptualization, funding acquisition, writing – original draft, writing – review and editing, supervision.

## Conflicts of Interest

The authors declare no conflicts of interest.
